# Rethinking Carbohydrate Intake and Time in Range in Children and Adolescents with Type 1 Diabetes

**DOI:** 10.3390/nu13113869

**Published:** 2021-10-29

**Authors:** Valentino Cherubini, Monica Marino, Marco Marigliano, Claudio Maffeis, Angela Zanfardino, Ivana Rabbone, Sara Giorda, Riccardo Schiaffini, Antonella Lorubbio, Serena Rollato, Antonio Iannilli, Dario Iafusco, Andrea E. Scaramuzza, Renee Bowers, Rosaria Gesuita

**Affiliations:** 1 Department of Women’s and Children’s Health, G. Salesi Hospital, 60123 Ancona, Italy; valentino.cherubini@gmail.com (V.C.); Antonio.Iannilli@ospedaliriuniti.marche.it (A.I.); 2 Pediatric Diabetes and Metabolic Disorders Unit, University of Verona, 37100 Verona, Italy; marco.marigliano@univr.it (M.M.); claudio.maffeis@univr.it (C.M.); 3 Regional Center of Pediatric Diabetology, University of Campania “L. Vanvitelli”, 80100 Naples, Italy; angela.zanfardino@unicampania.it (A.Z.); serenarollato@gmail.com (S.R.); dario.iafusco@unicampania.it (D.I.); 4 Department of Health and Science, University of Oriental Piedmont, 28100 Novara, Italy; ivana.rabone@uniupo.it; 5 Department of Medical Sciences, University of Turin, 10092 Turin, Italy; saragiorda.91@gmail.com; 6 Diabetes Unit, Pediatric Hospital Bambino Gesù, 00031 Roma, Italy; riccardo.schiaffini@gmail.com (R.S.); antonella.lorubbio@opbg.net (A.L.); 7 Division of Pediatrics, Pediatric Diabetes, Endocrinology and Nutrition, ASST Cremona, 26100 Cremona, Italy; a.scaramuzza@gmail.com; 8 Population Health, Faculty of Health Sciences, University of Ottawa, 64 Sherbrooke Avenue, Ottawa, ON 61350, Canada; renee.bowers13@gmail.com; 9 Center of Epidemiology and Biostatistics, Polytechnic University of Marche, 60123 Ancona, Italy; r.gesuita@staff.univpm.it

**Keywords:** time in range, macronutrients, children and adolescents, type 1 diabetes, multi-center observational study, continuous glucose monitoring

## Abstract

The aim of this study was to evaluate the association between macronutrient intake and time in range (TIR) of 70–180 mg/dL in children and adolescents with type 1 diabetes (T1D). A multi-center study recruited patients with T1D using continuous glucose monitoring (CGM) between January 2019 and January 2020 from centers across Italy. Diet intake was recorded using three-day weighed food diaries. Nutrients were evaluated as percentages of total intake. TIR was considered at target if the percentage of readings was higher than 70%. Clinical and nutritional factors associated with TIR at target were analyzed using multiple correspondence analysis and multiple logistic regression. Data from 197 participants (53% male, median age 11.6 years, median HbA1c 55.2 mmol/mol, median TIR 60%) were analyzed. Macronutrient intake was 45.9% carbohydrates, 16.9% protein, 37.3% fat, and 13.1 g/day fiber (median values). TIR > 70% was observed in 28% of participants; their diet contained more protein (17.6%, *p* = 0.015) and fiber (14.4 g/day, *p* = 0.031) than those not at target. The probability of having a TIR > 70% was significantly higher with 40–44% consumption of carbohydrates compared with 45–50% consumption of carbohydrates and with the use of a carbohydrate counting system. Based on these results, a five percent reduction in the percentage of carbohydrate intake can help children and adolescents with T1D achieve the goal of a TIR > 70%. Both a lower and higher percentage of carbohydrate intake appears to reduce the probability of reaching the target TIR > 70%. These results require validation in other populations before being used in clinical practice.

## 1. Introduction

Medical nutrition therapy is a cornerstone of diabetes management and includes education on carbohydrate estimation as part of an overall eating pattern to improve health outcomes [1]. Monitoring carbohydrate intake, whether by carbohydrate counting or experience-based estimation, remains a key strategy in achieving glycemic control [2]. Nevertheless, it has been shown that the dietary intake of young people with type 1 diabetes (T1D) in the community generally fails to meet recommended nutrient intakes as outlined in the International Society for Pediatric and Adolescent Diabetes (ISPAD) dietary guidelines for T1D [3,4]. In a large cohort of young people with T1D, the overall intake of total and saturated fats was high, while intake of fruits, vegetables, and grains was low [5]. Cross-sectional studies on children with T1D have suggested that diets characterized by lower fat [6], lower added sugar [7], higher carbohydrates [8], higher fiber, and higher fruit and vegetable consumption [6,7,8] are associated with lower glycated hemoglobin (HbA1c). Nutrition analysis in patients with T1D is relevant, because both overall diet quality and macronutrient distribution are associated with improved glycemic control [9]. Measurement of HbA1c is currently the most important method used to evaluate glycemic control, but continuous glucose monitor (CGM)-based percentage of time spent in the target range 70–180 mg/dL (TIR) has been considered an emerging measure of glycemic control because it is positively associated with reduced micro and macrovascular complications [10,11]. The use of both non-automated [12] and automated diabetes technologies such as closed loop control systems (CLC) have been shown to improve TIR [13]. The aim of this study was to analyze the association between clinical factors and macronutrient intake with CGM-based TIR in children and adolescents aged 2–17 years in the real-world community setting.

## 2. Materials and Methods

Pediatric diabetes centers with experience in the use of CGM and the availability of a specialized pediatric dietitian in childhood diabetes were invited to participate in this multi-center study. Five centers met these criteria and agreed to participate (Torino, Verona, Ancona, Roma, and Napoli). Inclusion criteria for participants were a diagnosis of T1D for over six months, between the ages of 2–17 years, using Dexcom G6 CGM System (Dexcom, Inc., San Diego, CA, USA) for over six months, having an active connection using Clarity^®^ software (Dexcom international Switzerland, Horw), an HbA1c ≤ 10% (86 mmol/L) during the three months prior to recruitment, and parents available to collect and record nutritional information for three days. To avoid potential discrepancies due to different accuracies of various CGMs, only patients using a Dexcom G6 were recruited. Exclusion criteria were unwillingness to use the CGM, use of a CGM other than Dexcom G6, unwillingness to share glucose data with the center, unavailability or inability of the parents to collect nutritional data, diagnosis of celiac disease, use of predictive low glucose management or CLC system, and HbA1c > 10% before the study. This study was approved by the Independent Ethics Committees of all five participating centers.

### 2.1. Study Procedures

From January 2019 to January 2020, before the start of the SARS-CoV-2 outbreak in Italy, all participants meeting the eligibility criteria were invited to participate in the study during their scheduled annual visit. All participating parents and adolescents over the age of 14 provided written informed consent. At the annual visit, a qualified researcher collected demographic information and clinical data on diabetes management. Additionally, parents had previously given written permission to connect their child’s Dexcom G6 blood glucose sensor (Dexcom international Switzerland, Horw) to the cloud connected to the pediatric diabetes centers. During the annual visit, this link was verified. Finally, a blood sample was added to the clinical examination to study T1D complications.

### 2.2. Nutritional Assessment

Dietary intake was assessed using a three-day weighed dietary record. A trained dietitian in each center advised families that the main purpose of the study was to analyze TIR with usual daily intake of food, so participants were advised to maintain all their current daily eating habits during the study. A kitchen scale (Soehnle Digital) and a food diary were provided. Parents of patients included in the study were carefully instructed on how to collect data and were provided with instructions on how to evaluate foods and record data using the validated DONALD study’s three-day weighed food diary [14]. Parents’ ability to measure nutrient intake was verified by the dietitian by way of a practical test before the study. They were asked to collect data in the food diary for three days (Sunday, Monday, and Tuesday) of the two weeks following recruitment. Through the food diary, they provided information about type and brand names of food items, time and location of eating, and recipes. For commercial food items, the packages or the food labels were collected and the product information was added to the dietary record using Winfood^®^ software (Medimatica, Teramo, Italy). Semi-quantitative recording was allowed when weighing was not possible, e.g., eating meals or snacks away from home.

### 2.3. Variables

Clinical and demographic characteristics included date of birth and date of diabetes diagnosis, gender, weight, height, number of episodes of severe hypoglycemia or ketoacidosis in the last 12 months, glycated hemoglobin (HbA1c), lipid profile (total cholesterol, HDL cholesterol, LDL cholesterol, triglycerides), weekly hours of physical activity, type of insulin therapy (MDI or CSII), average total daily insulin dose during the preceding week, and the use of a carbohydrate counting system. All centers used the same analytical laboratory methods. Total cholesterol, HDL cholesterol, and triglycerides were measured in stored plasma samples by an enzymatic method using a Beckman Coulter Olympus AU 480 (Beckman Coulter, Brea, CA, USA), and LDL was calculated using the Friedewald equation, which includes total cholesterol, HDL cholesterol, and triglycerides. HbA1c was measured with the DCA Vantage^®^ Analyzer.

Continuous glucose monitor-based glucose metrics (TIR, percentage of time < 54 mg/dL, 54–70 mg/dL 180–250 mg/dL, and >250 mg/dL, coefficient of variation, percentage of time CGM was active) were collected during the 15 days following the recruitment visit in which the participants’ food diaries were collected.

Food diaries were coded and quantified by the same dietitian (MM) for all centers. The dietary intake was assessed by calculating the total daily kilocalorie intake and the percentages of sugars, total carbohydrates, saturated fatty acids, monounsaturated fatty acids, polyunsaturated fatty acids, and protein intake in diets. The amount of daily cholesterol (g/day) and daily fiber (g/day) were also assessed as part of the dietary intake.

### 2.4. Statistical Analysis

The Shapiro-Wilk test results showed that quantitative variables were not normally distributed, so a non-parametric approach was used for the analysis.

The characteristics of the study sample were evaluated according to the percentage of time with glucose between 70 and 180 mg/dL (TIR ≤ 70% and >70%). Quantitative variables were summarized using medians and interquartile ranges and qualitative variables as absolute and percentage frequencies. Group comparisons between those with TIRs > 70% and those with TIRs ≤ 70% were performed using the Wilcoxon-Mann-Whitney test in the case of quantitative variables and the chi-squared test or Fisher’s exact test (when expected frequencies were < 5) for qualitative variables.

Multiple correspondence analysis (MCA), an exploratory statistical technique, was used to detect all the characteristics common to participants with T1D who had a percentage TIR > 70%. Metabolic control (HbA_1c_ grouped as either <7% and ≥7% [53 mmol/mol]), insulin delivery system (CSII or MDI), use of the carbohydrate counting system (yes or no), percentage of total carbohydrate (CHO: <40, 40–44, 45–50, >50), protein (P: <15, 15–20, >20), and fat (<35, ≥35), and TIR levels were analyzed simultaneously. MCA organizes the modalities of categorical variables into a multiple contingency table to calculate row and column frequencies. The frequencies are then projected onto a Cartesian plane to obtain a graphical representation of the associations between the variables. The modalities that are close to each other are those shared by the same patients, and the groupings of the modalities allow the interpretation of associations between the variables. The overall variability explained by the MCA model is indicated by the inertia, assuming a value equal to 100% if the model is able to explain all the variability.

Multiple logistic regression analysis was used to estimate the independent effect of patients’ clinical and nutritional characteristics on the probability of having a TIR > 70%. All estimates were obtained calculating 95% confidence intervals (CIs). The likelihood ratio (LR) test and Hosmer-Lemeshow test were used to select the most parsimonious model and to evaluate the model’s goodness of fit.

A probability < 0.05 was used to assess statistical significance, and all statistical analyses were performed using R version 4.0.4.

## 3. Results

A total of 197 children and adolescents were enrolled in this study. The overall median TIR was 60% (IQR 47–71%). The clinical characteristics and CGM-based glucose metrics of the participants according to the TIR cut-off are shown in Table 1. Fifty-five participants [27.9% (95% CI 21.8–34.7)] reported a TIR > 70%, with a median score of 77 (IQR 70–82).

Participants with TIR > 70% had significantly shorter disease duration and lower HbA1c levels. No significant differences were found between the two groups in terms of other clinical and lipid characteristics. Moreover, a TIR > 70% was significantly associated with lower percentage of time in hyperglycemia (% time 180–250 mg/dL and >250), higher percentage of time in hypoglycemia (% time 54–70 mg/dL), lower % CV, and higher percentage of time with the CGM active.

The nutritional profiles of participants according to TIR levels were also evaluated (Table 2). Patients with a TIR > 70% consumed a significantly higher percentage of protein and fiber per day. No significant differences were found between the two groups with respect to the consumption of other nutrients. Overall, 17 participants (8.6%) met all the three macronutrient ISPAD goals [15], with no significant difference between patients with a TIR > 70% or TIR ≤ 70%. Participants with a TIR > 70% more frequently consumed a carbohydrate percentage between 40% and 44% and a protein percentage of >20%. A protein percentage < 15% was significantly more frequent in participants with a TIR ≤ 70%. No significant differences were found between the two groups when considering overall fat, saturated fat, and fiber targets separately.

**Figure 1:**
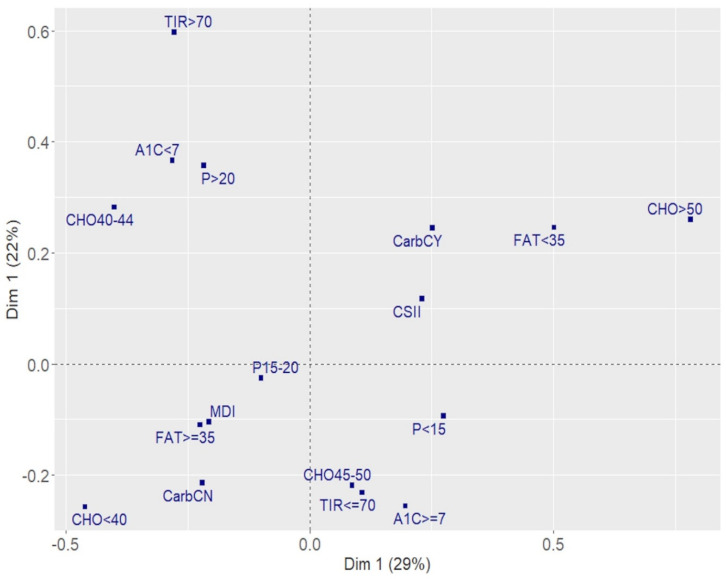
Associations between nutrients and metabolic control. Results of the multiple correspondence analysis. A1c > 7: HbA1c > 7% (53 mmol/mol); A1c < 7: HbA1c ≤ 7% (53 mmol/mol); CSII: insulin pump; MDI: multiple daily injection; CarbCN: no use of a carbohydrate counting system; CarbCY: use of a carbohydrate counting system; CHO < 40: % of total carbohydrate less than 40%; CHO40-44: between 40% and 44%; CHO45-50: between 45% and 50%; CHO > 50: greater than 50%; FAT < 35: % of fat <35%; FAT ≥ 35: % of fat ≥ 35%; P < 15: % of protein less than 15%; P15–20: between 15% and 20%; P > 20: greater than 20%; TIR > 70: % time 70–180 mg/dL more than 70%; TIR ≤ 70: % time 70–180 mg/dL up to 70%.

Figure 1 shows the results of the MCA. The two identified dimensions explained 51% of the total variability. It was possible to identify four groups, as follows:

(1) The first group in the second quadrant (II) contained participants with a TIR > 70% with optimal metabolic control. They consumed a protein percentage > 20% and carbohydrate between 40% and 44%.

(2) The opposite quadrant (IV) contained participants that did not have good metabolic control (HbA_1c_ ≥ 7% [53 mmol/mol] and TIR ≤ 70%). They consumed a low protein percentage (<15%) and a carbohydrate percentage between 45% and 50%.

(3) The first quadrant (I) contained participants using the carbohydrate counting system and CSII, and they consumed > 50% carbohydrate and a low percentage of fat (<35%).

(4) The third quadrant (III) contained participants characterized by a protein percentage between 15% and 20%, a high percentage of fat (≥35%), and a low percentage of carbohydrate (<40%), treated with MDI, and not using the carbohydrate counting system.

Quadrants II and IV identified a factorial plane in which patients consuming less carbohydrate and more protein with respect to ISPAD guidelines and at metabolic target were opposite patients characterized by poor metabolic control consuming the recommended percentage of carbohydrate as per ISPAD guidelines.

Table 2 shows the results of multiple logistic regression analysis adjusting for age, diabetes duration, insulin dose (units/kg/day), and kcal/kg/day intake. The probability of having a TIR > 70% was significantly higher with a percentage carbohydrate consumption between 40% and 44% compared with a percentage of 45–50% and with the use of a carbohydrate counting system. The longer the disease duration, the lower the probability of having a TIR > 70%; the higher the age, the lower the units of insulin dose/kg; and the lower the energy intake (kcal/kg) per day, the higher the probability of having a TIR > 70%.

## 4. Discussion

To our best knowledge, this is the first study reporting associations between TIR ≥ 70% and macronutrient intake in children and adolescents with T1D. Our results showed that participants consuming a diet with a carbohydrate percentage of 40–44% were more frequently at target for TIR and HbA1c. Moreover, the probability of being at target was significantly higher when compared to those with carbohydrate intake of 45–50% (Table 3).

The median TIR of the entire cohort did not reach the established upper target of greater than 70%, as suggested for patients with T1D [15]; however, it reached the minimum criterion of TIR at 60% if the HbA1c goal is 7.5%, as indicated for young people under the age of 25 years.

Our results showed that the probability of reaching a TIR > 70% increased with age. This is consistent with American Diabetes Association recommendations indicating that glycemic goals may need to be modified to consider the fact that younger children may have a form of “hypoglycemic unawareness”, in that they lack the cognitive capacity to recognize and respond to hypoglycemic symptoms and may be at greater risk of sequelae from hypoglycemia. In addition, children below the age of 5 years may be at risk of permanent cognitive impairment after episodes of severe hypoglycemia [16]. Therefore, more effort is needed to improve TIR in younger children by increasing parents’ diabetes education, refining insulin treatment [17], or using more advanced technological systems such as closed-loop control, which reduces the occurrence of severe hypoglycemia [18,19].

It is also noteworthy that participants’ body weights and lipid profiles were within the normal range for age and gender. The lipid profile of our series was comparable to a previous report of Italian children with type 1 diabetes [6] showing a mean LDL of 92 mg/dL and HDL of 58.5 mg/dL; in our study, no difference was detected in LDL and HDL values between participants achieving a TIR > 70% and those who did not.

Importantly, only 8.6% of all participants met current ISPAD macronutrient recommendations for all components, with no significant differences between those who achieved a TIR > 70% and those who did not. On the other hand, only 38.6% and 31.0% of all participants consumed carbohydrates and fats according to ISPAD guidelines, respectively.

To date, the association between macronutrient distribution and glycemic control has mainly focused on HbA1c [4,6,7,8,20], which is the best known marker of complication risk and commonly assessed quarterly as suggested by diabetes clinical practice guidelines. On the other hand, the use of HbA1c alone for assessing glycemic control can be misleading [20], as the same HbA1c value can be associated with good, fair, or even poor glycemic control as judged by the different potential mean glucose levels calculated by the CGM. In addition, observational studies analyzing macronutrient intake have usually been based on a three-day food diary record, while HbA1c reflects blood glucose concentrations over 3–4 months. As macronutrient intake may vary over the 3–4-month period, the association between HbA1c and macronutrient intake can be unreliable.

CGM-based glycemic metrics allow for the relationship between nutritional intake and glycemic pattern to be measured over a short period of time such as two weeks or even less. Hence, CGM metrics may be more helpful in diabetes management with regards to the type and quantity of macronutrients consumed.

While several nutritional factors may influence metabolic control, carbohydrate intake is one of the primary determinants of postprandial glucose levels. Results from the Diabetes Complication and Control Trial (DCCT) showed that participants randomly assigned to intensive therapy with a dietary intake higher in fat and saturated fat and lower in carbohydrate had worse glycemic control [20]. On the other hand, results from a longitudinal study reported that the higher the carbohydrate intake, the more the HbA1c increased [21]. The recommended carbohydrate intake in the 2014 ISPAD guidelines [22] of 50–55% of total energy intake was reduced in the 2018 ISPAD guidelines [1] to 45–50%. To assess the percentage of carbohydrate intake associated with the best metabolic control as measured by TIR, we considered four classes. Using the 45–50% class as a reference, we found that the probability of having an TIR > 70% increased more than 2.5 times if carbohydrate intake was between 40–44%. Moreover, patients consuming a carbohydrate percentage of 40–44% more frequently had an HbA1c < 7% (Figure 1). In addition, the carbohydrate counting system was associated with a higher probability of TIR > 70% (Table 3) and it was used more frequently by participants consuming a carbohydrate percentage > 50% (Figure 1). The use of the carbohydrate counting system therefore represents a key strategy for improving metabolic control and reaching the target TIR. Its use should continue to be recommended in the daily management of diabetes.

In our analysis, the overall sugar intake among all participants was lower than that reported in previous studies [6,10,23], with no difference between participants who achieved or did not achieve the target TIR. However, it should be noted that, due to the study design, the amount of sugar used to correct hypoglycemia could not be analyzed. Participants achieving a TIR > 70% consumed more fiber per day (Table 2); however, the impact of this nutrient was not significant when considered in the multiple analysis.

Previous studies would have used traditional blood glucose monitoring and not a real time CGM device. A recent study using data from the DCCT showed that decreased TIR was strongly associated with the risk of microvascular complications and, although HbA1c remains a key outcome measure, TIR can add value as an outcome measure [23]. As a result of the widespread use of CGM and increased accuracy of results, we can now more widely use TIR as a measure in clinical practice. The results of our study need confirmation in a larger sample and in populations that have different dietary intakes. If confirmed, these data could have implications in clinical practice, because small changes in diet could increase the percentage of TIR in children and adolescents with T1D.

Our study has a few limitations. The cross-sectional design did not allow us to prospectively assess an association between macronutrients and metabolic control. Also, the inclusion of patients using an insulin pump system based on different algorithms did not allow for an analysis of the effect of technologies on the probability of achieving target TIR. Evaluating the effect of the macronutrient composition in patients using the same algorithm remains an area of investigation. Nevertheless, this study had many strengths including a multi-center design, data collected from many participants with well-characterized T1D, and use of the same analytical laboratory methods and food consumption measure. There was consistency in the training for parents, and the macronutrient intake calculations were conducted by the same dietitian for all the centers involved. Moreover, parents’ ability to measure nutrient intake was tested before the study, which helped to eliminate any potential errors in assessing food consumption. Finally, there were no potential conflicts of interest with Dexcom regarding the use of this glucose sensor that affected the study protocol or results.

## 5. Conclusions

This study shows that a small reduction in the percentage of carbohydrate intake to 40–44%, compared to the current ISPAD guidelines which indicate 45–50%, improves the percentage of TIR. This allows children and adolescents with type 1 diabetes to achieve the goal of a TIR greater than 70%. Additionally, both a lower and higher percentage of carbohydrate intake than the ISPAD guidelines appears to reduce the probability of reaching the target TIR by 70%.

It should be noted that increasing the TIR requires a multifactorial approach that takes into account modifiable factors such as macronutrient intake, type of treatment, insulin titration, physical activity as well as non-modifiable factors such as age, diabetes duration, and experienced hypoglycemia unawareness.

These findings require validation in other populations before they can be incorporated into clinical practice guidelines and remain an area of future research. Further studies are necessary to investigate TIR and macronutrients in children from different socioeconomic and ethnic backgrounds.

## Figures and Tables

**Table 1:** Clinical characteristics according to percentage of time in range.

	Total	% Time 70–180 mg/dL		
	(*n* = 197)	≤70 (*n* = 142)	>70 (*n* = 55)	*p*-Value
Clinics				
Sex, F	92 (46.7)	64 (45.1)	28 (50.9)	0.564
Age, years	11.6 (8.6; 14.3)	11.4 (8.2; 14)	11.9 (9.5; 15)	0.069
Pubertal stage, Tanner 2–5	119 (60.4)	82 (57.7)	37 (67.3)	0.287
Insulin delivery System, CSII	93 (47.2)	69 (48.6)	24 (43.6)	0.641
Diabetes duration, years	3.7 (2; 6.8)	4.7 (2.5; 7.3)	2.1 (1.1; 5.5)	0.001
BMI SDS	0.2 (−0.5; 0.7)	0.2 (−0.4; 0.7)	−0.1 (−0.6; 0.7)	0.469
Physical activity, hours/w	3 (2; 4)	3 (1; 4)	3 (2; 5)	0.150
HbA1c, %	7.2 (6.5; 7.7)	7.4 (6.9; 7.9)	6.4 (6; 6.7)	<0.001
Total cholesterol, mg/dL	155 (140; 175)	157 (142; 176)	153 (135; 174)	0.304
Cholesterol HDL, mg/dL	59 (49; 71)	58 (49; 71.2)	60 (48; 70)	0.723
Cholesterol LDL, mg/dL	88.4 (73; 103)	89 (73.3; 102.6)	84 (72.5; 103)	0.666
Triglycerides, mg/dL	53 (43; 67)	53.5 (43; 67.2)	51 (43.5; 63)	0.771
Carb counting system, Yes	92 (46.7)	60 (42.3)	32 (58.2)	0.064
CGM-based glucose metrics				
% time <54 mg/dL	0.2 (0; 0.9)	0.2 (0; 0.9)	0.3 (0; 0.9)	0.552
% time 54–70 mg/dL	2 (0.5; 4.5)	1.5 (0.4; 4)	3 (1.4; 5.2)	0.010
% time 180–250 mg/dL	37 (24; 50)	43 (35; 56)	17.9 (12.2; 23)	<0.001
% time >250 mg/dL	10 (4; 20)	14.5 (9.1; 25.4)	2.2 (0.7; 3.8)	<0.001
% CV	36 (32; 41)	37.6 (32.9; 41)	33.6 (30.2; 36.9)	<0.001
% time CGM active	95 (89; 98)	93.1 (85.7; 97.6)	97.3 (93.8; 98.7)	<0.001

Values are presented as median (IQR) or *n* (%); *p*-values refer to Wilcoxon rank-sum test or chi-square test.

**Table 2:** Subjects’ nutritional profiles according to percentage of time in range.

	**Total**	**% Time 70–180 mg/dL**		
	**(*n* = 197)**	**≤70 (*n* = 142)**	**>70 (*n* = 55)**	***p*** **-Value**
Nutrients [median (IQR)]				
Kcal/day	1668 (1495–1943)	1675 (1496–1940)	1615 (1488–1943)	0.689
Protein	16.9 (14.4; 19)	16.3 (14.1; 18.3)	17.6 (15.8; 19.4)	0.015
Carbohydrate	45.9 (42.3; 49.1)	46.2 (42.4; 49.1)	43.4 (41.5; 48.2)	0.098
Fat	37.3 (33.3; 41.0)	37.6 (33.4; 37.2)	37.2 (33.2; 41.2)	0.750
SFA	9.6 (7.8; 10.9)	9.5 (7.8; 10.7)	9.1 (7.6; 10.9)	0.753
PUFA	10.3 (7; 14.9)	11.2 (7; 15.1)	9.2 (6.3; 13.3)	0.196
MUFA	16.4 (13.8; 19.4)	16.1 (13.7; 19.1)	17.4 (15; 20.3)	0.211
Sugar	11.1 (8.2; 13.6)	11.2 (8.2; 13.7)	11.1 (8.6; 12.7)	0.741
Fiber (g/day)	13.1 (10.2; 16.3)	12.8 (9.6; 15.7)	14.4 (11.7; 17.1)	0.031
ISPAD nutritional goals, *n* (%)				
All macronutrient goals	17 (8.6)	11 (7.7)	6 (10.9)	0.572
CHO <40%	31 (15.7)	24 (16.9)	7 (12.7)	0.615
40–44%	53 (26.9)	29 (20.4)	24 (43.6)	0.002
45–50%	76 (38.6)	60 (42.3)	16 (29.1)	0.124
>50%	37 (18.8)	29 (20.4)	8 (14.5)	0.457
FAT <35%	61 (31.0)	41 (28.9)	20 (36.4)	0.309
Saturated fat <10%	110 (55.8)	78 (54.9)	32 (58.20)	0.750
Protein <15%	60 (30.5)	50 (35.2)	10 (18.2)	0.031
15–20%	113 (57.4)	79 (55.6)	34 (61.8)	0.531
>20%	24 (12.2)	13 (9.2)	11 (20.0)	0.031
Fiber ≥ age (years) + 5	54 (27.4)	37 (26.1)	17 (30.9)	0.483

All macronutrient goals refer to meeting goals for carbohydrate, fat, and protein simultaneously. Values are presented as median (IQR) or n (%); p-values refer to the Wilcoxon rank-sum test or chi-squared test. Abbreviations: P: protein; SFA: saturated fatty acid; PUFA: polyunsaturated fatty acids; MUFA: monounsaturated fatty acids.

**Table 3:** Factors associated with target TIR (>70%).

Variable	OR	90%CI	*p*-Value
Total CHO (<40% vs. 45–50%)	0.2	0.04–0.81	0.033
Total CHO (40–44% vs. 45–50%)	2.56	1.05–6.37	0.039
Total CHO (>50% vs. 45–50%)	0.73	0.23–2.19	0.577
P (15–20% vs. <15%)	1.78	0.73–4.58	0.213
P (>20% vs. <15%)	2.3	0.54–9.81	0.256
CHO counting (yes vs. no)	2.29	1.05–5.12	0.039
Diabetes duration (years)	0.76	0.65–0.88	<0.001
Age (years)	1.31	1.14–1.53	<0.001
Insulin dose (units/kg/day)	0.02	0.00–0.15	<0.001
BMI SDS	0.79	0.51–1.23	0.299
Kcal/day	0.99	0.98–1.00	0.664

Results from logistic regression analysis; LR test: χ^2^ with 11 df, χ^2^ = 60.14, *p* < 0.001; Hosmer and Lemeshow goodness of fit test: χ^2^ with 8 df, χ^2^ = 7.47, *p* = 0.487.

## Data Availability

Data is contained within the article.
